# The role of satisfaction with care among factors affecting health-related quality of life in people with cancer: a cross-sectional study

**DOI:** 10.3389/fonc.2025.1712478

**Published:** 2026-01-26

**Authors:** Federica Sancassiani, Goce Kalcev, Mirian Agus, Olga Mulas, Elena Massa, Diego Primavera, Giulia Origa, Mariarita Monni, Veronica Vacca, Francesca Pibiri, Antonio Egidio Nardi, Clelia Madeddu, Giovanni Caocci, Mario Scartozzi, Marco Cruciata, Rosangela Caruso, Maria Giulia Nanni, Martino Belvederi Murri, Luigi Grassi, Mauro Giovanni Carta

**Affiliations:** 1 Department of Medical Sciences and Public Health, University of Cagliari, Cagliari, Italy; 2 Center of Liaison Psychiatry and Psychosomatics, University Hospital of Cagliari, Cagliari, Italy; 3 Department of Pedagogy, Psychology, Philosophy, University of Cagliari, Cagliari, Italy; 4 Hematology and Stem Cell Transplantation Center, Hospital “Businco”, Azienda Ospedaliera “Brotzu”, Cagliari, Italy; 5 Laboratory of Panic and Respiration, Institute of Psychiatry of Federal University of Rio de Janeiro (IPUB/UFRJ), Rio de Janeiro, Brazil; 6 Institute of Psychiatry, Department of Neuroscience and Rehabilitation, University of Ferrara, Ferrara, Italy; 7 Hospital University Psychiatry Unit, S. Anna Hospital/Local Health Trust, Ferrara, Italy; 8 PhD Program Tropical Medicine and Department of Nursing, Universidad Popular del Cesar, Valledupar, Colombia

**Keywords:** cancer, health-related quality of life, oncology, psychosomatics, satisfaction with care, psychoncology

## Abstract

**Background:**

Despite advances in therapies that have increased the duration of post-diagnosis survival, living with long-term adverse effects of cancer and its treatment is common. This study aims to evaluate satisfaction with care (SC) as a dimension affecting Health-Related Quality of Life (HRQoL) among people with cancer, considering other sociodemographic and clinical variables supposedly impacting HRQoL.

**Methods:**

This cross-sectional study used an *ad hoc* form to collect sociodemographic and clinical variables. The SF-12 and TPQ were used to evaluate HRQoL and SC, respectively. The relation between SC, socio-demographic, clinical variables (predictors), and HRQoL (criterion) was assessed using hierarchical linear regression, controlling for age, cancer stage, and time of care.

**Results:**

263 patients (49.8% males; age 61.2 ± 13.8 years) from two cancer units. Positive correlations (p<0.05) between SC and HRQoL. Females have a poorer HRQoL than males (ß= .416, CI 95% [.162;.671], p=0.001), as well as patients from the hospital ward compared to those in the day hospital service (ß= -.459, CI 95% [-.782 -.137], p=0.005). Greater SC referred to the service features predict better HRQoL (ß= .338, CI 95% [.154;.523], p<0.001).

**Conclusions:**

Given the cross-sectional design, causal inferences cannot be drawn; however, identifying satisfaction with care and other factors associated with HRQoL among people with cancer may help inform prevention and rehabilitation programs.

## 1 Introduction

The management and organization of cancer care represent one of the most pressing public health challenges worldwide. Cancer is among the leading causes of death globally, and cancer-related mortality is projected to more than double by 2060, largely due to population aging and increased exposure to lifestyle-related risk factors such as physical inactivity, excess body weight, and environmental stressors, including noise, light, and air pollution. These conditions, already prevalent in high-income countries, are becoming increasingly widespread in low- and middle-income countries, particularly in urbanized areas (1–3).

Advances in early detection and treatment have significantly improved survival rates, yet many individuals continue to experience long-term impairments and persistent symptoms due to the disease and its treatments (4). Surgical complications, fatigue, pain, and long-lasting side effects such as fear of recurrence and emotional distress often compromise survivors’ daily functioning and well-being, even after achieving remission (5–8). These challenges are particularly pronounced among younger patients, for whom a cancer diagnosis may trigger long-term psychological distress and a marked decline in health-related quality of life (HRQoL) (9, 10).

In line with the World Health Organization’s definition of health as “a resource for everyday life, not the objective of living,” encompassing physical, psychological, and social capacities (11), the concept of HRQoL has emerged as a critical outcome measure in oncology (12). HRQoL is a multidimensional construct comprising physical, mental, and social dimensions and reflects the individual’s perception of health in relation to disease, treatment, and survivorship (13, 14). Numerous studies have shown that HRQoL in people with cancer is influenced by sociodemographic and clinical variables, such as age, sex, cancer site and stage, treatment type, duration of care, and the presence of adverse effects (15, 16). Recent evidence also confirms the role of age, tumor characteristics, functional impairments and psychosocial distress in shaping HRQoL across different cancer populations (5–8, 17).

Among the factors impacting HRQoL, patient satisfaction with care (SC) has gained increasing attention. First introduced by the World Health Organization in 1989 as a core component of healthcare quality assurance, SC is defined as the degree to which care meets the individual needs and expectations of patients, typically assessed through subjective evaluations of service quality (18). In oncology, SC serves as a key indicator of care quality, reflecting how well clinical services align with patient-centered values and preferences (19–22). Recent literature highlights how models of care delivery, follow-up pathways and supportive care resources influence both patient satisfaction and subjective health outcomes (23–25).

Although it is well established that higher HRQoL can enhance patient SC by fostering greater engagement, autonomy, and trust throughout the cancer care continuum (21, 22, 26–28), less attention has been paid to the reverse relationship. Specifically, the extent to which SC, particularly its organizational and interpersonal dimensions, may independently contribute to better perceived HRQoL has not been sufficiently explored. While several studies have documented associations between clinical outcomes (i.e.: fatigue, functional status) and patient SC (28, 29), few have isolated the unique role of SC beyond medical and demographic factors as a modifiable predictor. Furthermore, existing literature tends to focus on either clinical effectiveness or psychosocial predictors of HRQoL, often overlooking the patient’s subjective care experience as a potentially modifiable factor. This gap is particularly relevant in oncology, where patients routinely undergo frequent medical visits, diagnostic procedures, and complex treatment regimens. In such a setting, the quality of interactions with healthcare providers and the organization of care may play a pivotal role in shaping patients’ perceptions of their well-being and quality of life (29).

This study aims to address this gap by examining whether SC serves as a determinant of HRQoL in people with cancer, after accounting for sociodemographic and clinical variables. Clarifying this relationship may have important implications for improving HRQoL through patient-centered service delivery.

## 2 Materials and methods

### 2.1 Study design

This is a cross-sectional study.

### 2.2 Setting

From 2018 to 2020, consecutive people with cancer of both the inpatient and outpatient clinics at the Oncology Unit, University Hospital of Cagliari “Policlinic – Duilio Casula” and the Day-Hospital Service of the Hematology Unit and Stem Cell Transplantation Center, Hospital “Businco”, Azienda Ospedaliera Brotzu, Cagliari, Italy, were invited to participate in the study. Data collection was discontinued at the end of the recruitment period due to the onset of the COVID-19 pandemic; however, this did not influence the cross-sectional data included in the present analyses. Subjects who signed the informed consent were recruited to establish the study cohort.

### 2.3 Participants and recruitment

The sample was selected based on the following inclusion criteria: ≥ 18 years old, male/female sex, histologically confirmed diagnosis of malignant neoplasm in active treatment (surgery, chemotherapy, radiation therapy, immunotherapy, hormone therapy).

The exclusion criteria were: non-acceptance to participate in the study (non-signing the informed consent), less than 18 years old.

### 2.4 Variables

In this study, perceived health-related quality of life (HRQoL) is considered the primary outcome. The selected predictors include age, gender, hospital, type of oncology service, adherence to cancer treatment, duration of care, cancer stage, treatment-related toxicity, and patient satisfaction with care, evaluated concerning both staff performance and the organizational characteristics of the oncology services. Age, cancer stage and time of care were supposed to potentially confound the association between HRQoL and SC (15, 16).

### 2.5 Study tools

- An *ad hoc* report was used to collect the socio-demographic and clinical-oncological data, specifically: gender, age, marital status, employment status, educational level, kind of service, follow-up timing, kind of cancer, cancer stage, toxicity of treatments, intent of treatment, response to treatment, and adherence at 3 months of follow-up.
- The SF-12 (Short Form Health Survey – 12 item) (30), in the Italian version (31) was administered to evaluate HRQoL. It is a self-report questionnaire that consists of 12 items investigating two dimensions, linked to perceived physical and mental health. The total score ranges from 12 to 47, with higher scores indicating a higher HRQoL. According to population studies, a score <36 indicates low perceived HRQoL levels (32).
- The TPQ (Treatment Perception Questionnaire) (33, 34) in the Italian version (35) was used to evaluate satisfaction with care. The Italian version of the TPQ had been previously adapted for routine use in oncology services. Its psychometric validation (35) was conducted concurrently on the same cohort of patients included in this study and published shortly after the end of the data collection period, confirming its reliability and stability. It is a self-reported questionnaire that includes 10 items and considers two dimensions: the first one regards the perception of patients towards the nature and extent of their contact with the health staff (5 items); the second regards aspects of the care program and its procedures and regulations (5 items). Each item is recorded using a five-point Likert scale (strongly agree – strongly disagree). The total scoring range of the TPQ is 0–40, with higher scores indicating greater overall satisfaction with care, while higher scores on each subscale reflect greater satisfaction with staff-related and service-related features, respectively.

### 2.6 Risk of bias

To minimize potential sources of bias, participants were consecutively recruited from both inpatient and outpatient oncology settings, regardless of cancer type or stage, and data were collected using validated self-report instruments (SF-12 and TPQ). Nonetheless, several sources of bias may persist. Selection bias cannot be entirely ruled out due to the non-probabilistic sampling strategy, which may have favored individuals more motivated or able to participate. Additionally, information bias may arise from the self-administered nature of the questionnaires, which are susceptible to social desirability and recall effects. Finally, although partial correlation and hierarchical regression models were used to adjust for known confounders (age, cancer stage, time of care), residual confounding by unmeasured psychological or social variables remains possible.

### 2.7 Sample size

This study adopted an observational, cross-sectional design without a pre-specified sample size calculation. The observational nature of the study and the consecutive recruitment approach did not allow for an *a priori* sample size determination, which may limit statistical power for subgroup analyses. Recruitment was based on a consecutive sampling strategy over a defined period (2018–2020) in two oncology centers, and the final sample (n = 263) reflects the total number of eligible patients who consented to participate during this timeframe.

Based on *post hoc* considerations, the available sample size was adequate to detect medium effect sizes in hierarchical multivariable linear regression models. Assuming an alpha level of 0.05, a sample size of 263 provides approximately 80% statistical power to detect effect sizes in the range of f² ≈ 0.10–0.15 in models including up to 10 predictors, as applied in the present analyses.

### 2.8 Ethical aspect

The study protocol received approval from the Ethical Committee of Azienda Ospedaliero-Universitaria di Cagliari, Italy, in 2018 (number PG/2018/13269). A written informed consent, after being presented with comprehensive explanations of the objectives and methodologies of the study, informed on data security, and assured of their right to discontinue their participation at any point, was requested from each participant. All procedures were conducted in accordance with the Helsinki Declaration (36).

### 2.9 Statistical methods

Data were analyzed using the open-source software Jamovi (version 2.3.0). Frequencies and percentages or mean ± standard deviation were used for descriptive statistics of sociodemographic and clinical-oncological variables. Partial linear correlations were computed between all the dimensions of HRQoL and satisfaction with care, controlling for age, cancer stage, and time of care.

A hierarchical linear regression (Enter method) was performed to assess the role of satisfaction with care-related variables in predicting patients’ HRQoL. Specifically, the total score of the SF-12 was used as the criterion, as it provides a global measure of health-related quality of life, consistent with the study aim of examining overall associations between satisfaction with care and HRQoL. Separate analyses of the physical and mental component scores were not conducted to avoid model over-fragmentation and because domain-specific effects were beyond the primary scope of the study.

Following the Enter method, two blocks of analysis were specified. Block 1 included age, gender, hospital, kind of service, adherence to cancer treatment, time of care, cancer stage, and treatment toxicity. Block 2 additionally included the two dimensions of satisfaction with care assessed by the TPQ questionnaire, referring to staff-related and service-related features, respectively. Multicollinearity was assessed according to commonly accepted thresholds. Missing data were handled using a complete-case (listwise deletion) approach; no imputation methods were applied. All statistical tests were considered significant at p < 0.05.

## 3 Results

### 3.1 Characteristics of the study cohort

Two hundred and sixty-three people with cancer (n= 131 males, 49.8%; age: mean ± sd= 61.2 ± 13.8 years, range= 19–86 years) were recruited by a non-probabilistic sampling from the two Italian hospitals (Businco n=62, 23.6%; Policlinico n=201, 76.4%). 76.4% and 23.6% of the participants had solid or blood cancer, respectively, and the more prevalent treatment intent was non-curative (66.1%). The descriptive data of assessed variables were detailed in **Table 1**.

**Table 1:** Characteristics of the study cohort.

Variables	Overall (N = 263)
HOSPITAL	
Hospital Businco	62 (23.6%)
Policlinic – Duilio Casula	201 (76.4%)
GENDER	
M	131 (49.8%)
F	132 (50.2%)
AGE	
Mean (SD)	61.2 (13.6)
Range	19.0 - 86.0
MARITAL STATUS	
single	55 (20.9%)
married	177 (67.3%)
divorced	10 (3.8%)
widow	21 (8.0%)
EMPLOYMENT STATUS	
retired	108 (41.1%)
employed	81 (30.8%)
housewife	46 (17.5%)
unemployed	25 (9.5%)
student	3 (1.1%)
EDUCATIONAL LEVEL	
< primary school	1 (0.4%)
primary school	32 (12.2%)
secondary school	93 (35.4%)
high school	100 (38.0%)
military academy	1 (0.4%)
university degree	32 (12.2%)
higher	4 (1.5%)
KIND OF SERVICE	
Day Hospital	217 (82.5%)
Hospital Ward	46 (17.5%)
TIME OF CARE	
(Missing)	1 (0.4%)
first visit	9 (3.4%)
<6 months	102 (38.8%)
6–12 months	48 (18.2%)
>12 months	103 (39.2%)
CANCER SITE	
gastroenteric	92 (35%)
gynecological	32 (12.2%)
breast	32 (12.2%)
lung	18 (6.9%)
uro-genital	17 (6.5%)
head and neck	1 (0.4%)
lymphoid neoplasms	44 (16.7%)
myeloid neoplasms	10 (3.8%)
rare	9 (3.4%)
other	3 (1.1%)
Unknow	5 (1.9%)
CANCER KIND	
blood	62 (23.6%)
solid	201 (76.4%)
CANCER STAGE*	
1	5 (1.9%)
2	24 (9.1%)
3	38 (14.4%)
4	196 (74.5%)
TOXICITY**	
(Missing)	4 (1.5%)
0	76 (28.9%)
1	50 (19.1%)
2	59 (22.4%)
3	33 (12.5%)
4	28 (10.7%)
5	13 (4.9%)
TREATMENT INTENT	
Radical (curative, adjuvant, neo-adjuvant)	84 (32%)
Non-curative (palliative, maintenance)	174 (66.1%)
Support (not specified)	5 (1.9%)
RESPONSE TO TREATMENT	
ongoing evaluation	62 (23.6%)
absence of cancer	56 (21.3%)
cancer in progress	50 (19%)
not applicable	1 (0.4%)
partial	28 (10.6%)
stable	65 (24.7%)
(Missing)	1 (0.4%)
ADHERENCE TO CANCER TREATMENT***	
not evaluated	7 (2.7%)
YES	230 (87.5%)
NO	26 (9.9%)

*1= unique localization in one nodal station or extra-nodal; 2= two or more localizations from the same side of the diaphragm, 3= localizations from both sides of the diaphragm; 4= diffuse disease.

**From 0 (absence) to 5 (death), according to Common Toxicities Criteria (CTC), version 4.0 (37).

***Assessed by the treating oncologist based on clinical records and treatment attendance.

### 3.2 Partial correlations between HRQoL and satisfaction with care

Partial linear correlations between the psychological dimensions assessed by the SF-12 (HRQoL) and the TPQ (satisfaction with care), controlling the relations for the variables “age”, “cancer stage” and “time of care”. **Table 2** shows that there are significant positive correlations between all the dimensions related to satisfaction with care and HRQoL, except for TPQ “Staff” and SF-12 “Physical”.

**Table 2:** Partial linear correlations between HRQoL and satisfaction with care in the cohort (N = 263).

	SF-12 PHYSIC		SF-12 MENTAL		TPQ STAFF		TPQ SERVICE		SF-12 TOTAL		TPQ TOTAL	
SF12 PHYSIC	—											
	—											
SF-12 MENTAL	0.589	***	—									
	< .001		—									
TPQ STAFF	0.081 (ns)		0.172	**	—							
	0.195		0.005		—							
TPQ SERVICE	0.186	**	0.245	***	0.757	***	—					
	0.003		< .001		< .001		—					
SF-12 TOTAL	0.858	***	0.914	***	0.159	*	0.259	***	—			
	< .001		< .001		0.010		< .001		—			
TPQ TOTAL	0.144	*	0.224	***	0.933	***	0.941	***	0.225	***	—	
	0.020		< .001		< .001		< .001		< .001		—	

Controlling for ‘AGE’, ‘CANCER STAGE’, and ‘TIME OF CARE’; *p <.05, **p <.01, ***p <.001; ns=not significant.

### 3.3 The impact of satisfaction with care and other variables on HRQoL

To evaluate the effect of the variables on the patients’ HRQoL, hierarchical linear Regression (Enter method) was applied, using the SF-12 “Total” score (HRQoL) as the criterion, the socio-demographic and clinical variables as predictors in Block 1. Then, in Block 2, the dimensions of satisfaction with care related to “staff” and “service” as evaluated by the TPQ were added (see **Table 3**). All variables included in the model met the assumptions for linear regression within acceptable ranges, indicating no relevant multicollinearity among predictors.

**Table 3:** Hierarchical linear regression (enter method). Criterion variable: SF-12 Total – Block 2.

Predictor	Estimate	SE	t	p	Stand. estimate	95% confidence interval	
						Lower	Upper
AGE	-0.0312	0.0316	-0.9863	0.325	-0.0660	-0.198	0.0659
GENDER							
F – M	2.6738	0.8291	3.2249	0.001	0.4169	0.162	0.6715
HOSPITAL							
Policlinico – Businco	0.9428	1.7261	0.5462	0.585	0.1470	-0.383	0.6771
KIND OF SERVICE							
Hospital Ward – Day Hospital	-2.9479	1.0503	-2.8068	0.005	-0.4596	-0.782	-0.1370
ADHERENCE TO CANCER TREATMENT***							
not evaluated – YES	-0.4602	3.9733	-0.1158	0.908	-0.0718	-1.292	1.1486
NO – YES	-0.6580	1.3663	-0.4816	0.631	-0.1026	-0.522	0.3171
TIME OF CARE							
<6 months – first visit	1.7736	2.1762	0.8150	0.416	0.2765	-0.392	0.9449
6–12 months – first visit	2.8424	2.2703	1.2520	0.212	0.4432	-0.254	1.1405
>12 months – first visit	3.1191	2.2241	1.4024	0.162	0.4863	-0.197	1.1694
CANCER STAGE*							
2 – 1	0.7110	3.0528	0.2329	0.816	0.1108	-0.827	1.0485
3 – 1	-1.2639	2.9707	-0.4254	0.671	-0.1971	-1.109	0.7154
4 – 1	-2.3611	2.8927	-0.8163	0.415	-0.3681	-1.257	0.5203
TOXICITY**							
1 – 0	0.7466	1.1258	0.6632	0.508	0.1164	-0.229	0.4622
2 – 0	1.5141	1.1011	1.3750	0.170	0.2361	-0.102	0.5743
3 – 0	0.0995	1.4713	0.0676	0.946	0.0155	-0.436	0.4674
4 – 0	-2.0421	2.0464	-0.9979	0.319	-0.3184	-0.947	0.3101
5 – 0	1.2585	2.5195	0.4995	0.618	0.1962	-0.578	0.9701
**TPQ STAFF**	-0.1903	0.1699	-1.1203	0.264	-0.1043	-0.288	0.0791
**TPQ SERVICE**	0.5729	0.1586	3.6120	< .001	0.3389	0.154	0.5238

*1= unique localization in one nodal station or extra-nodal; 2= two or more localizations from the same side of the diaphragm, 3= localizations from both sides of the diaphragm; 4= diffuse disease.

**from 0 (absence) to 5 (death), according to Common Toxicities Criteria (CTC), version 4.0 (37).

***Assessed by the treating oncologist based on clinical records and treatment attendance.

In this regression, at Block 1 the R2 adjusted was 0.039 [F (17;241) =1.63, p=.058] without any significance. The data regarding Block 1 of this analysis were included in the **Supplementary Materials**. However, at Block 2, which included the two dimensions of TPQ, a significant increment of the R2 adjusted, 0.1027 [F (19;239 = 2.55, p<.001], was observed. Examining this second block, “gender” results as a significant predictor (Beta=.416, CI 95% [.162;.671], p=0.001), highlighting that females have a lower total score in SF-12 (poorer HRQoL) than males. Furthermore, there is a significant effect of “kind of service” (Beta= -.459, CI 95% [-.782 -.137], p=0.005), because the patients in the hospital ward have a lower total score in SF-12 (poorer HRQoL) than patients in the day hospital service. Finally, a significant effect of the scale of TPQ “Service” (Beta=.338, CI 95% [.154;.523], p<0.001) was observed, so that higher scores in this dimension are related to higher total scores in SF-12 (better HRQoL). The TPQ “Staff” dimension had no significant effect on the total score of SF-12. The detailed results of this analysis are shown in **Table 3**.

## 4 Discussion

This study confirms the well-established association between satisfaction with care (SC) and health-related quality of life (HRQoL) among individuals with cancer. However, while previous research has often emphasized the unidirectional impact of HRQoL on SC, our findings suggest that higher levels of SC, particularly regarding the organizational features of oncology services, are also significant predictors of improved HRQoL. This insight underscores the bidirectional nature of the relationship between SC and HRQoL and highlights the clinical relevance of patient-centered service design. Recent evidence further supports this relationship, showing that patient-reported experiences with care, including satisfaction, perceived quality of interactions, and the adequacy of supportive resources, are significant determinants of QoL across the cancer trajectory (24, 38).

Cancer poses a substantial physical and psychological burden, with treatments frequently leading to functional limitations and long-term consequences that affect various life domains (39, 40). Even among disease-free survivors, persistent effects of the illness and treatment may compromise well-being (41). Understanding the factors that shape HRQoL is therefore essential not only for clinical practice but also for informing health policy. As previously reported (5–7, 10), people with cancer typically experience lower HRQoL than the general population. Moreover, poor HRQoL among patients with hematological cancers appears comparable to that observed in patients with solid tumors or other chronic conditions, such as major depression and carotid atherosclerosis (10).

Consistent with previous studies (42–45), SC was positively associated with all dimensions of HRQoL, after adjusting for age, cancer stage, and treatment duration, except in the case of physical HRQoL. This suggests that physical domains (i.e.: pain and functional limitations) are more directly influenced by the disease and its medical management, rather than by patients’ interactions with the care team.

A notable finding of this study is that SC related to the organizational aspects of care, but not to interactions with clinical staff, was a significant predictor of HRQoL. While positive staff interactions generally enhance patient engagement and trust, the lack of significance observed in our cohort may reflect the predominance of outpatient participants (82.5%), who typically experience shorter and less continuous contact with care professionals. In such contexts, patients may rely more heavily on the predictability, clarity, and efficiency of service organization, which may explain why the “Service” dimension of the TPQ more strongly predicted HRQoL. Additionally, measurement-related factors should be taken into account. The staff-related dimension of the TPQ may have shown limited variability in this sample, potentially due to uniformly high ratings or ceiling effects, which could have reduced its ability to detect associations with HRQoL. Moreover, staff satisfaction may influence HRQoL indirectly, through mechanisms such as treatment adherence or perceived continuity of care, which were not fully captured in the present analysis.

Our findings also add nuance to sociodemographic influences. Unlike prior studies showing that younger patients experience lower HRQoL (15, 40, 46–48), no age-related associations were detected in our sample. This may be attributed to the high prevalence of advanced-stage cancer (74.5%), potentially obscuring age effects due to the overwhelming burden of diffuse disease. Conversely, consistent with existing literature (15, 49, 50), female participants reported significantly lower HRQoL. This could be explained by sex-specific cancer types such as gynecological and breast cancers, which are frequently treated with chemotherapy and associated with fatigue, sleep disturbances, and affective symptoms (51, 52).

As expected, patients receiving inpatient care reported significantly lower HRQoL compared to those attending day hospitals, possibly reflecting more severe clinical conditions requiring hospitalization and the greater impact of illness on daily functioning.

Importantly, the predictive value of sociodemographic and clinical factors on HRQoL became significant only after accounting for SC in the hierarchical regression model. Among the domains of SC, aspects related to service organization, such as the availability of information on treatment decisions, time allocated to addressing patient concerns, satisfaction with sessions, and alignment between expected and received support, emerged as strong predictors of HRQoL. These findings highlight the need for oncology care systems to prioritize procedural transparency and responsiveness to patient needs.

From a clinical perspective, these results point out the importance of designing and implementing survivorship care plans that address late and long-term effects, psychosocial needs, lifestyle recommendations, and follow-up care (44, 53). Equally crucial is integrating HRQoL and other patient-reported outcomes into routine clinical assessments. Enhanced patient–physician communication, facilitated by appropriate training, is known to improve outcomes by aligning care with patient values and perceptions. Recent literature shows that patient-reported experiences are increasingly recognized as actionable indicators that can inform personalized survivorship planning (23–25). Our results support this trend by highlighting how organizational aspects of care can serve as modifiable targets to improve patient outcomes.

Moreover, given the persistent challenges faced by cancer survivors, innovative and targeted preventive and rehabilitative interventions are warranted. Virtual Reality (VR) and repetitive Transcranial Magnetic Stimulation (rTMS) are two promising tools with demonstrated potential in improving physical, emotional, and neurobiological outcomes in oncology settings, with high levels of patient satisfaction with these interventions (54–57), suggesting both their acceptability and their relevance as components of supportive cancer care. The relevance of this research direction is supported by ongoing clinical trials, such as the one registered under ClinicalTrials.gov ID: NCT06589544 (58).

This study presents several limitations that should be acknowledged when interpreting the findings. First, the use of a non-probabilistic sampling strategy may introduce selection bias, as participation was limited to patients who were available and willing to provide consent. Consequently, the results may not be generalizable to the broader population of individuals with cancer. Second, no formal sample size calculation was performed before recruitment. Because the study relied on consecutive recruitment over a predefined period, a pre-specified sample size calculation was not feasible, which may have limited statistical power for subgroup analyses. Although the sample was sufficiently large to detect moderate associations, the study may have been underpowered to explore subgroup differences or to detect small effect sizes, limiting the robustness of some inferences. Third, data on SC and HRQoL were collected through self-administered instruments, which are susceptible to reporting biases such as social desirability or momentary emotional influences. Fourth, the sample was recruited from two oncology services located in a single geographic area in Southern Italy. Contextual and organizational characteristics specific to these settings may have influenced patient perceptions and experiences, thereby limiting the external validity and generalizability of the findings to other regions or healthcare contexts. Fifth, although the regression models adjusted for several clinical and demographic covariates, important psychosocial variables such as anxiety, depression, coping strategies, social support, and socioeconomic status were not assessed. The omission of these variables may have resulted in residual confounding, potentially biasing the estimated associations between satisfaction with care and HRQoL. Therefore, the observed relationships should be interpreted with caution, as unmeasured psychosocial factors may partially account for the reported effects. Finally, the cross-sectional nature of the study precludes conclusions regarding causality and directionality, as well as temporal ordering between satisfaction with care and HRQoL. Longitudinal research is needed to clarify the directionality and potential mediating pathways of these associations over time.

## 5 Conclusions

This study provides compelling evidence for the bidirectional relationship between satisfaction with care (SC) and health-related quality of life (HRQoL) in individuals with cancer, emphasizing the pivotal role of service-related factors in shaping patient well-being. While disease stage and treatment burden remain key determinants of physical HRQoL, our findings underscore the independent and significant contribution of patient-perceived care quality, particularly in terms of service organization, to overall HRQoL.

These insights call for a paradigm shift in oncology practice, where clinical outcomes are measured not only by survival or symptom management but also by how well care aligns with patient expectations and experiences. Incorporating SC and HRQoL as standard outcome measures can help develop more holistic, patient-centered care models. Furthermore, in light of the persistent physical and psychological challenges faced by cancer survivors, the implementation of tailored preventive and rehabilitative strategies becomes essential. Emerging interventions such as Virtual Reality (VR) and repetitive Transcranial Magnetic Stimulation (rTMS) offer promising avenues for enhancing post-treatment recovery, quality of life, and satisfaction with care. Their inclusion in future survivorship care frameworks may help address unmet needs and improve long-term outcomes.

To advance this field, longitudinal studies with more diverse samples and comprehensive psychosocial assessments are warranted. These should aim to unravel the complex interactions between clinical, psychological, and service-related variables, and to inform supportive care in oncology.

## Data Availability

The datasets presented in this article are not readily available because the EU individual privacy rules. Requests to access the datasets should be directed to Prof. Federica Sancassiani, federicasancassiani@yahoo.it.
