# Supplementary material for: The role of satisfaction with care among factors affecting health-related quality of life in people with cancer: a cross-sectional study
Source: Front Oncol. 2026 Jan 26;15:1712478. doi: 10.3389/fonc.2025.1712478 (PMC12883354; doi:10.3389/fonc.2025.1712478)
Supplement: Supplementary file 1 [file Table1.docx]

**Supplementary materials:**

Table S1: Hierarchical Linear regression (Enter method). Criterion variable: SF-12 Total – Block 1

|  | | |  | | | 95% Confidence Interval | |
| --- | --- | --- | --- | --- | --- | --- | --- |
| **Predictor** | **Estimate** | **SE** | **t** | **p** | **Stand. Estimate** | **Lower** | **Upper** |
| **AGE** | -0.0313 | 0.0326 | -0.9607 | 0.338 | -0.0664 | -0.2024 | 0.0697 |
| **GENDER** |  | |  |  |  |  |  |
| F – M | 2.3646 | 0.8542 | 2.7683 | **0.006** | 0.3687 | 0.1063 | 0.6310 |
| **HOSPITAL** |  |  |  |  |  |  |  |
| Policlinico – Businco | 0.1706 | 1.7760 | 0.0961 | 0.924 | 0.0266 | -0.5189 | 0.5721 |
| **KIND OF SERVICE** |  |  |  |  |  |  |  |
| Hospital Ward – Day Hospital | -3.1280 | 1.0830 | -2.8883 | **0.004** | -0.4877 | -0.8203 | -0.1551 |
| **ADHERENCE**  **TO CANCER TREATMENT***** |  |  |  |  |  |  |  |
| not evaluated – YES | 0.3195 | 4.1062 | 0.0778 | 0.938 | 0.0498 | -1.2113 | 1.3109 |
| NO – YES | -0.7493 | 1.4090 | -0.5318 | 0.595 | -0.1168 | -0.5496 | 0.3159 |
| **TIME OF CARE** |  |  |  |  |  |  |  |
| <6 months – first visit | 2.5971 | 2.2428 | 1.1580 | 0.248 | 0.4049 | -0.2839 | 1.0938 |
| 6-12 months – first visit | 3.1107 | 2.3478 | 1.3250 | 0.186 | 0.4850 | -0.2361 | 1.2061 |
| >12 months – first vistit | 3.3916 | 2.2982 | 1.4758 | 0.141 | 0.5288 | -0.1770 | 1.2346 |
| **CANCER STAGE*** |  |  |  |  |  |  |  |
| 2 – 1 | 1.9927 | 3.1310 | 0.6364 | 0.525 | 0.3107 | -0.6509 | 1.2723 |
| 3 – 1 | 0.2306 | 3.0451 | 0.0757 | 0.940 | 0.0360 | -0.8993 | 0.9712 |
| 4 – 1 | -0.5447 | 2.9502 | -0.1846 | 0.854 | -0.0849 | -0.9910 | 0.8212 |
| **TOXICITY**** |  |  |  |  |  |  |  |
| 1 – 0 | 0.8243 | 1.1636 | 0.7084 | 0.479 | 0.1285 | -0.2289 | 0.4859 |
| 2 – 0 | 1.9973 | 1.1330 | 1.7629 | 0.079 | 0.3114 | -0.0366 | 0.6594 |
| 3 – 0 | -0.1387 | 1.5210 | -0.0912 | 0.927 | -0.0216 | -0.4888 | 0.4455 |
| 4 – 0 | -1.9938 | 2.1157 | -0.9424 | 0.347 | -0.3109 | -0.9606 | 0.3389 |
| 5 – 0 | 0.2412 | 2.5892 | 0.0931 | 0.926 | 0.0376 | -0.7576 | 0.8328 |
|  |  |  |  |  |  |  |  |

*1= unique localization in one nodal station or extra-nodal; 2= two or more localizations from the same side of the diaphragm, 3= localizations from both sides of the diaphragm; 4= diffuse disease.

** from 0 (absence) to 5 (death), according to Common Toxicities Criteria (CTC), version 4.0 [37].

*** Assessed by the treating oncologist based on clinical records and treatment attendance.
